# Exploring Motivation and Barriers to Physical Activity among Active and Inactive Australian Adults

**DOI:** 10.3390/sports5030047

**Published:** 2017-06-28

**Authors:** Erin Hoare, Bill Stavreski, Garry L. Jennings, Bronwyn A. Kingwell

**Affiliations:** 1 Metabolic and Vascular Physiology, Baker Heart and Diabetes Institute, Melbourne, VIC 3004, Australia; Garry.Jennings@baker.edu.au (G.L.J.); Bronwyn.Kingwell@baker.edu.au (B.A.K.); 2 National Heart Foundation of Australia, Melbourne, VIC 3000, Australia; Bill.Stavreski@heartfoundation.org.au; 3 Sydney Medical School, University of Sydney, Camperdown, NSW 2006, Australia

**Keywords:** physical activity, motivation, barriers, Australia, population health

## Abstract

Physical inactivity is a major global public health issue associated with a range of chronic disease outcomes. As such, the underlying motivation and barriers to whether or not an individual engages in physical activity is of critical public health importance. This study examines the National Heart Foundation of Australia Heart Week Survey conducted in March 2015. A total of 894 (40% female) Australian adults aged 25–54 years completed the survey, including items relating to motivation and barriers to being physically active. The most frequently selected responses regarding motivation for physical activity among those categorised as active (*n* = 696) were; to lose or maintain weight (36.6%, 95% CI 33.1–40.3), avoid or manage health condition (17.8%, 95% CI 15.1–20.8), and improve appearance (12.8%, 95% CI 10.5–15.5). Some gender differences were found with a greater proportion of females (43.8%, 95% CI 38.0–49.8) reporting lose or maintain weight as their main motivation for being physically active compared to males (31.9%, 95% CI 27.7–36.6). Among those categorised as inactive (*n* = 198), lack of time (50.0%, 95% CI 43.0–56.8) was the most frequently reported barrier to physical activity. While empirical studies seek to understand the correlates and determinants of physical activity, it is critical that beliefs and perceptions enabling and prohibiting engagement are identified in order to optimise physical activity promotion in the community.

## 1. Introduction

Physical inactivity is highly prevalent globally and is associated with a range of chronic disease outcomes, including type 2 diabetes, coronary heart disease, some cancers, and shortened life expectancy [1,2]. A major global analysis revealed that in 2013 the economic burden of physical inactivity cost international health-care systems approximately (INT$) 53.8 billion [3]. With known negative health consequences and the significant global economic burden of physical inactivity, it is concerning that the Australian Health Survey 2014–2015 revealed that close to half (45%) of Australian adults aged 18–64 years did not participate in physical activity at levels recommended by current guidelines (150 to 300 min of moderate intensity or 75 to 150 min vigorous intensity each week) in the week prior to surveying [4]. Additional evidence suggests that the levels of daily time spent sedentary are increasing due to technological advances and environmental changes where sitting has become the default behaviour in the office, transport and domestic environments [5]. While the health consequences of physical inactivity are well-documented, identifying the enabling and impeding factors driving physical activity is a critical step to achieving sustained behavioural change and reduction in the attributable disease burden.

There is consensus that a broad range of factors influence physical activity, spanning psychological, environmental, social, and policy domains [6,7]. Individual psychological factors such as confidence and perceived competence predict physical activity participation [8]. Additionally, home and social environment including emotional and logistical support, as well as environmental factors such as crime-related safety and access to physical activity spaces (i.e., footpaths, parks and green spaces), all modulate activity [9]. A recent summary of interventions to improve population-level physical activity levels suggests greater effectiveness in socio-ecological approaches which target multiple levels of influence, many of which are beyond the health system [10]. Given this evidence, motivation for engagement in physical activity must be examined in the complex and multi-dimensional context in which such behaviours occur.

Evidence from self-determination theory (SDT), based on the interplay between intrinsic and extrinsic motives in driving behaviour [11], has consistently shown that identification (e.g., consciously valuing a goal) and intrinsic motivations (e.g., succeeding at challenges, experiencing enjoyment) predicts physical activity [8]. Further studies have investigated self-reported motivation, although studies primarily focused on children/adolescents [12], young adults in university settings [13], or specific adult groups such as those with chronic illness [14,15,16]. Few studies have examined Australian adults, with evidence limited to breast cancer survivors [17], older adults [18], physically inactive [19], socio-demographically disadvantaged [20], and low quality study design such as small sample size [21]. One study [22] examined barriers, enjoyment and preference of activity among Australian adults, but predominately focused on differences in activity levels, and was conducted over a decade ago.

In the context of current national and international efforts to increase population-wide physical activity levels, the primary aim of this study was to explore the barriers and motivators for physical activity in a large, Australian adult sample.

## 2. Materials and Methods

This study examines data from the National Heart Foundation of Australia Heart Week Survey conducted in March 2015. The survey was developed by the National Heart Foundation of Australia. Participants were recruited via an online market research company and were recruited to meet quotas for age, gender and area of residence to reflect the wider Australian population (further information available from authors). Participants had no association with the Heart Foundation nor was any reference to the Heart Foundation made in the survey invitation. All variables were checked for missing data and in all cases there was less than 10%; case-wise deletion was used. 

Participants reported their current physical activity levels, categorised into either little-to-no activity (inactive) or some-to-regular physical activity (active). Those categorised as inactive were asked to report their main barriers to physical activity, and those categorised as active were asked to report their main motivations for physical activity. The item relating to motivation for physical activity allowed one single response whereas multiple responses were possible for barriers to physical activity. This was to gain a comprehensive understanding of the various barriers to physical activity, in order to inform public health messaging to increase activity among inactive adults. All items for categorising participants as active and inactive are reported in Supplementary Table S1. 

Analyses included descriptive statistics and proportions including 95% confidence intervals. Pearson Chi-Squared tests were used to examine differences between males and females, and differences in activity levels. Significance was assumed at *p* < 0.05. All data were analysed using Stata release V.14.1 (Stata Corp., College Station, TX, USA, 2013), and SPSS V.22 (IBM Corp. Released 2013. IBM SPSS Statistics for Windows, Version 22.0. IBM Corp: Armonk, NY, USA).

## 3. Results

### 3.1. Participant Characteristics

A total of 894 adult participants had complete data available and were included in the analysis. Just over half of the total sample (*n* = 531, 59.4%) were male, and approximately a third made up each age bracket 25–34 years (*n* = 278, 31%), 35–44 years (*n* = 335, 38%), and 45–54 years (*n* = 281, 31%). Half (*n* = 465, 52%) had tertiary education. A total of 528 (59%) participants were overweight or obese, and 366 (41%) were normal weight based on self-reported height and weight, and using World Health Organization criteria whereby a body mass index (BMI) >25 kg/m^2^ is overweight and equal to or greater than 30 kg/m^2^ is considered obese [23]. Proportions relating to area of residence (not reported, available from authors) of the participating sample were representative of the wider Australian population [24]. 

When active and inactive groups were compared, there was a significant difference among males aged 25–34 years, with a greater proportion classified as inactive compared to active. When gender differences were compared, significant differences were found between those aged 25 to 34 years and classified as active, with a lower proportion of males compared to females represented in this group. A significantly greater proportion of overweight/obese males were represented in the active category compared to overweight/obese females, and a greater proportion of normal weight females were categorised as active compared to their normal weight male counterparts.

### 3.2. Active vs. Inactive

More than three quarters of the sample (*n* = 696, 78%) reported currently doing some physical activity (active group), although this might have been irregular. There were 22% (*n* = 198) who reported doing none to very little physical activity (inactive group). Participant characteristics stratified by the active and inactive category are reported in Table 1. The proportion of males and females categorised as active or inactive were similar (not reported). Half (50%) of the active group and 56% of the inactive group held tertiary education. Proportions of overweight/obesity were similar among the active (58%) and inactive (62%) groups. Very few gender differences were observed across active and inactive categories for age, education and weight status.

### 3.3. Motivations and Barriers for Physical Activity

Detailed information on responses is reported in Supplementary Table S1. The most frequently selected responses for physical activity motivation were to lose or maintain weight (36.6%, 95% CI 33.1–40.3), avoid or manage health condition (17.8%, 95% CI 15.1–20.8), and improve appearance (12.8%, 95% CI 10.5–15.5) (Table 2). Other motivations selected were to improve athletic performance and/or strength (11.5%, 95% CI 9.3–14.1), and improve mood (7.9%, 95% CI 6.1–10.2). A greater proportion of females (43.8%, 95% CI 38.0–49.8) selected lose or maintain weight compared to males (31.9%, 95% CI 27.7–36.6) and this difference was significant (*p* < 0.05). No other significant differences in responses between males and females were observed. Less than 4% selected options gain weight, improve focus, play with children/grandchildren, or to participate in social activities (Supplementary Table S1).

Among those categorised as inactive, lack of time was reported by half the sub-group as a barrier to physical activity (50.0%, 95% CI 43.0–56.8) and this was the most frequently selected response (Table 3). Other barriers selected were lack of enjoyment (43.9%, 95% CI 37.1–51.0) and preferring to do other things (42.9%, 95% CI 36.2–50.0). No significant differences were observed between males and females.

## 4. Discussion

While empirical studies seek to understand the correlates and determinants of physical activity, it is critical that beliefs and perceptions are valued as an important information source for physical activity promotion. Although limited by self-report and cross-sectional methodology, our study demonstrated the focus on weight- and appearance-related motivations among a large representative sample of Australian adults, and this was more common among females. Commonly perceived barriers to physical activity were lack of time, interest and enjoyment, and some participants identified social (nobody to exercise with) and self-esteem (lack of confidence) factors as barriers to being active.

Overweight/obesity in the current sample (59%) was reflective of objectively measured national estimates from the National Health Survey 2014–2015 which reported 63% of Australian adults aged 18 years and over were overweight/obese [4]. While three-quarters of the participating sample claimed to be active, given data on national activity levels [4], it is unlikely that this proportion met national guidelines for physical activity. Population estimates report that approximately half Australian of adults successfully achieve more than 150 min of moderate physical activity or more than 75 min of vigorous physical activity each week [4]. The likelihood of over reporting physical activity and underreporting anthropometry has previously been reported and it is possible that self-report bias may be present in these results. 

The finding that losing or maintaining weight was the most frequently reported motivation for physical activity is consistent with previous research suggesting physical appearance- and health-related motives for being active [25]. In relation to self-determination theory, Gavin et al. (2014) reported that motivations may reflect both intrinsic and extrinsic elements [25]. For example, weight-related motivations may reflect future and current health concerns which could be considered intrinsic, whereas a desire for physical attractiveness may refer to extrinsic motivations. Type of motivation aside, the finding that one-third of males and an even greater proportion of females identify losing or maintaining weight as their main motivation for physical activity does highlight the focus on weight-based drivers of behaviour, and this is an important consideration for potential leveraging in future interventions. 

A greater proportion of females compared to males reported losing or maintaining weight as the main motivation for physical activity, and also that a greater proportion of overweight/obese males were active compared to overweight/obese females. Body dissatisfaction has consistently been shown to be higher in females than males, and the internalisation of body ideals also differs by gender [26,27]. While females often report desired weight loss and thin ideals, males often report muscular body ideals. Body dissatisfaction relating to internalising ideals coincides with various psychological and physical health risks [28]. It is therefore conceivable that there is an interaction between such pressures, self-perceptions and the extent to which an individual engages in health behaviour. Such interactions, considered along with sociological and health behavioural theories, are important areas for future research.

Of the most frequently reported responses for motivation for physical activity, it is of particular interest that just 8% of the participating sample selected improve mood. The beneficial effects of physical activity, exercise and sport for prevention and treatment of mental health-related problems are well-documented [29,30,31]. While physical activity triggers release of endorphins and serotonin which are well documented to affect mood [32], it also provides opportunity to engage in social interaction, build self-efficacy, and protect against additional health conditions that co-occur with common mental disorders. The beneficial effects of physical activity for mood may form an important future national public health campaign, particularly given the alarming proportion of Australians suffering mood-related disorders, the inconsistent efficacy of pharmacological treatments, and the need for accessible and effective prevention methods.

The barriers to physical activity are consistent with previous literature reporting factors such as time availability, preference and lack of enjoyment as key constraints to activity, particularly among those who are inactive [22]. Public health strategies to overcome such barriers, such as time management and efforts to promote enjoyment such as social interaction and community connectedness, are critical to achieving national population activity recommendations. Given the large proportion of time Australian adults spend sedentary [4], and with increasing evidence of the physiological and metabolic benefits of breaking up sitting time [33,34,35], it is also recommended that integrating incidental physical activity into tasks which are habitual and/or enjoyable may assist in overcoming such barriers. Such strategies do not necessarily require great investment of time and can be accessible to a large proportion of adults. Lastly, it is noted that only a small (<15%) proportion of participants identified environmental factors (e.g., poor weather, lack of suitable facilities, traffic or road safety) as barriers, despite evidence demonstrating a relationship between such factors and physical activity [9]. While outside the scope of this current study, the environmental context of respondents would have provided valuable insight into the interactions between environment, individual perceptions and activity levels, and this is an important area for future research. 

This study was limited by the self-report methodology, particularly given known biases in reporting of health behavioural-related information [36,37]. While this study provided important insight into motivations for individuals who engage in physical activity, further insight would have emerged had those inactive also reported what they identify as physical activity motivation. Furthermore, future intervention opportunities may have emerged if participants had been asked to identify strategies to overcome barriers. With physical inactivity representing one of the largest modifiable contributors to global deaths and with the significant global economic burden of physical inactivity [3], what could potentially be harnessed to encourage physical activity is critically important for engaging and encouraging activity among this group. Including adults aged older than 54 years would have strengthened this study, in allowing comparisons to the nationally representative Australian Health Survey, and to provide insight into perceived barriers and motivators among older aged adults when physical activity typically declines [38]. Finally, the recruitment of participants was completed through an online market research company in partnership with National Heart Foundation of Australia. Although purposive sampling methods were designed to recruit participants who were representative of the wider Australian population, it is inevitable that their membership to the market research company may have led to biases in our results. 

## 5. Conclusions

This study provided insight into the motivations and barriers for physical activity among a large, Australian adult sample, representative of the wider population based on key health and demographic characteristics. The purpose of this study was to identify key drivers of behaviour, rather than critically appraise motivations and barriers, with the ultimate goal of exploring how to most effectively increase and maximise physical activity in the community. Weight-related motivations were widely reported among those active as key to engaging in physical activity behaviour, and this was found to be most frequent among females compared to males. The findings of this study may be used to inform intervention and prevention targets for this age group, particularly due to the national burden of disease attributable to physical inactivity, and the large proportion of Australian adults failing to achieve minimum activity recommendations. It is also recommended that gender-specific messages may be appropriate in promoting physical activity to achieve national targets. Mood- and mental health-related benefits of physical activity were infrequently reported as motivators for physical activity, and future initiatives may aim to raise awareness of such benefits. While limited by self-report and some survey design methodologies, this study provided valuable insight into the current physical activity-related perceptions of Australian adults. 

## Figures and Tables

**Table 1:** Participant demographic and health characteristics, categorised as active/inactive.

Participant Characteristics	Inactive ^a^ *n* = 198			Active ^b^ *n* = 696		
	Male *n* (%)	Female *n* (%)	Total *n* (%)	Male*n* (%)	Female *n* (%)	Total *n* (%)
***n***	109 (55.1)	89 (44.9)	198	422 (60.6)	274 (39.4)	696
**Age (years)**						
25–34	39 (35.8) *	28 (31.4)	67 (33.8)	102 (24.2) ^T^	109 (39.8)	211 (30.3)
35–44	43 (39.4)	38 (42.7)	81 (40.9)	167 (39.6)	87 (31.8)	254 (36.5)
45–54	27 (24.8)	23 (25.9)	50 (25.3)	153 (36.2)	78 (28.4)	231 (33.2)
**Level of education**						
Less than Year 12	7 (6.4)	10 (11.2)	17 (8.6)	28 (6.6)	18 (6.6)	46 (6.6)
Completed Year 12	11 (10.1)	6 (6.7)	17 (8.6)	58 (13.7)	29 (10.6)	87 (12.5)
Completed TAFE, Diploma or trade	25 (22.9)	28 (31.5)	53 (26.8)	116 (27.5)	93 (33.9)	209 (30.0)
Completed Tertiary	66 (60.6)	45 (50.6)	111 (56.0)	220 (52.2)	134 (48.9)	354 (50.9)
**BMI**						
Normal weight <25 kg/m^2^	32 (29.3)	43 (48.3)	75 (37.9)	143 (33.9) ^T^	148 (54.0)	291 (41.8)
Overweight/obese ≥25 kg/m^2^	77 (70.7)	46 (51.7)	123 (62.1)	279 (66.1) ^T^	126 (46.0)	405 (58.2)

^a^ Responded “little-to-no current physical activity”; ^b^ Responded “some-to-regular physical activity”; * Significant difference between inactive and active groups, within gender (*p* < 0.05); ^T^ Significant difference between males and females, within activity level (*p* < 0.05).

**Table 2:** Motivation for being physically active expressed as proportion of males and females who were categorised as active (*n* = 696).

Motivation	Male % (95% CI)	Female % (95% CI)	Total % (95% CI)	*p*
Which of the Following Is Your Main Motivation for Being Physically Active?				
Lose or maintain weight	31.9 (27.7–36.6)	43.8 (38.0–49.8)	36.6 (33.1–40.3)	*p < 0.05*
Avoid or manage health condition	18.7 (15.2–22.7)	16.4 (12.5–21.3)	17.8 (15.1–20.8)	*NS*
Improve athletic performance and/or strength	13.3 (10.3–16.9)	8.8 (5.9–12.8)	11.5 (9.3–14.1)	*NS*
Improve appearance	12.1 (9.3–15.5)	13.9 (10.2–18.5)	12.8 (10.5–15.5)	*NS*
Improve mood	8.3 (6.00–11.3)	7.3 (4.7–11.0)	7.9 (6.1–10.2)	*NS*
Other	15.6 (12.5–19.4)	9.9 (6.8–14.0)	13.4 (11.0–16.1)	*p < 0.05*

Significance testing between group differences for males and females (only one response allowed). *NS* = not significant.

**Table 3:** Barriers to physical activity for those who reported being inactive (*n* = 198) (multiple responses allowed) % proportion selected.

Barrier	Male	Female	Total	*p*
Which of the Following Are Your Main Barriers to Being Physically Active?				
Lack of time	46.8 (37.5–56.0)	53.9 (43.4–64.2)	50.0 (43.0–56.8)	*NS*
Prefer to do other things	38.5 (29.8–48.1)	48.3 (38.0–58.8)	42.9 (36.2–50.0)	*NS*
Lack of enjoyment	39.4 (30.6–49.0)	49.4 (39.0–59.9)	43.9 (37.1–51.0)	*NS*
Nobody to exercise with	17.4 (11.3–25.9)	21.3 (13.9–31.3)	19.2 (14.3–25.3)	*NS*
Lack of confidence	22.0 (15.1–30.9)	19.1 (12.1–28.8)	20.7 (15.6–27.0)	*NS*
Lack of money	14.7 (9.1–22.8)	17.9 (11.2–27.6)	16.2 (11.6–22.0)	*NS*

Note: further barriers were identified but not reported here due to the low proportion (<15%) of participants who selected them (data available from authors). *NS* = not significant.

## Supplementary Materials

The following are available online at http://www.mdpi.com/2075-4663/5/3/47/s1; Supplementary Table S1: Heart Week Survey active and inactive categories, items relating to motivations and barriers to physical activity with proportions
