# Supplementary material for: Exploring Motivation and Barriers to Physical Activity among Active and Inactive Australian Adults
Source: Sports (Basel). 2017 Jun 28;5(3):47. doi: 10.3390/sports5030047 (PMC5968958; doi:10.3390/sports5030047)
Supplement: Supplementary file 1 [file sports-05-00047-s001.pdf]

Supplementary Materials: Exploring Motivation and Barriers to Physical Activity among Active and Inactive Australian Adults

Erin Hoare [1,*], Bill Stavreski [2], Garry L Jennings [1,2,3,†] and Bronwyn A Kingwell [1,†]

Supplementary Table 1. Heart Week Survey active and inactive categories, items relating to motivations and barriers to physical activity with proportions

Item	Response	Category	n (%)
**All participants;**			894 (100)
**Which of the following best describes your current level of physical activity?**			
I do none or very little physical activity and I do not intend to start in the next six months		Inactive	52 (5.8)
I do none or very little physical activity, but I am thinking about starting in the next six months		Inactive	146 (16.3)
I do physical activity, but not regularly		Active	312 (34.9)
I do physical activity regularly but I only have begun to do so in the last six months		Active	119 (13.3)
I do physical activity regularly, and have done so for more than 6 months		Active	265 (29.6)
**If Active;**			696 (77.9)
**Which of the following is your main motivation for being physically active? (One response allowed)**			
Lose or maintain weight		Active	255 (36.6)
Gain weight		Active	7 (1.0)
Improve appearance		Active	89 (12.8)
Avoid or manage health conditions		Active	124 (17.8)
Improve mood		Active	55 (7.9)
Improve athletic performance and/or strength		Active	80 (11.5)
Improve focus		Active	10 (1.4)
Play with children/grandchildren		Active	18 (2.6)
Participate in social activities (i.e golf, tennis)		Active	23 (3.3)
Other		Active	35 (5.0)
**If Inactive;**		Active	198 (22.1)
**Which of the following are you main barriers to being physically active? (Multiple responses allowed)**			
Lack of time		Inactive	99 (50.0)
Prefer to do other things		Inactive	85 (42.9)
Ill health, injury or disability		Inactive	28 (14.1)
I feel too fat/overweight		Inactive	37 (18.7)
I do not enjoy exercise		Inactive	87 (43.9)
Lack of suitable local facilities		Inactive	11 (5.6)
I am too old		Inactive	13 (6.6)
Lack of money		Inactive	32 (16.2)
I have nobody to go with		Inactive	38 (19.2)
The weather puts me off		Inactive	32 (16.2)
Lack of confidence		Inactive	41 (20.7)
Lack of transport		Inactive	4 (2.0)
Traffic or road safety		Inactive	6 (3.0)
Lack of skills		Inactive	17 (8.6)
Nothing prevents me		Inactive	9 (4.6)
Other		Inactive	15 (8.0)
